# Inverse Vaccination for Autoimmune Diseases: Insights into the Role of B Lymphocytes

**DOI:** 10.3390/cells14141085

**Published:** 2025-07-16

**Authors:** Moncef Zouali

**Affiliations:** Graduate Institute of Biomedical Sciences, China Medical University, No. 91, Xueshi Road, North District, Taichung 404, Taiwan; moncef.zouali@wanadoo.fr

**Keywords:** regulatory B cells, regulatory T cells, autoimmunity, clinical trial, immune tolerance

## Abstract

A novel therapeutic approach, inverse vaccination, is being developed to combat autoimmune diseases and other inflammatory conditions. It aims to educate the immune system to recognize self-components as innocuous and stop reacting against them. Inverse vaccination, also referred to as tolerogenic vaccination, introduces autoantigens into the immune system to induce immune tolerance to the nominal antigen. In contrast to conventional vaccination, which aims to train the immune system to identify a pathogen as a potential threat that needs to be eradicated, inverse vaccination is designed to educate the immune system to recognize that an antigen is harmless and, consequently, extinguish the inflammatory attack of the tissues that contain the autoantigen. This article discusses recent progress in using inverse vaccination to design therapeutic interventions in several autoimmune diseases by deprivation of co-stimulatory signaling, tagging autoantigens to trigger immune tolerance in the liver, and mRNA vaccination. Also discussed is a tolerogenic feedback loop implicating B lymphocytes in inverse vaccination.

## 1. Introduction

Autoimmune diseases comprise a group of over 80 autoinflammatory disorders. In addition to well-known diseases, such as rheumatoid arthritis (RA), lupus, and multiple sclerosis (MS), other disorders, such as autoimmune encephalitis and gestational pemphigoid, are rare and difficult to diagnose, and patients may suffer for years before the disease is diagnosed [1]. The autoimmune attack may result in the abnormal growth of an organ or changes in its functions, and even in the destruction of body tissue. Their origin remains unclear, but genetic, epigenetic, and environmental factors have been identified [2]. Some of these diseases have no cure and require life-long treatments. Existing conventional therapies for autoimmune diseases include non-specific immunosuppression that necessitates long-term treatment and can result in undesirable side effects.

For reasons that remain under investigation, both central and peripheral tolerance are disrupted in autoimmune diseases, and the resulting auto-immune response attacks the body’s own components [3,4]. Abrogation of the resulting pathogenic autoreactivity by restoring immune tolerance to self-antigens would therefore block disease progression without hindering the immune system’s potential to respond to infectious threats. Rather than focusing on the consequences of the pathogenic process that underlies autoimmune disorders, an alternative strategy consists in mitigating the pathogenic processes involved through “tolerogenic vaccination”, which is also called “inverse vaccination”. Whereas conventional vaccination aims to trigger an immune response against the immunogen of interest and generate immune memory, tolerogenic vaccination strives to disrupt the inflammatory response and generate a state of immune tolerance [5]. It represents an opportunity to design specific treatments that are not associated with immunosuppression.

Specifically, inverse vaccination is an innovative immunotherapeutic approach aimed at inducing immune tolerance. In autoimmune diseases, inverse vaccination educates the immune system to tolerate specific self-antigens that are targeted by auto-reactive T and B lymphocytes as well as autoantibodies. Its goal is to suppress or to silence autoreactive immune cells, thereby preventing them from causing damage. Mechanistically, it aims to promote formation of regulatory T cells and/or regulatory B cells and secretion of anti-inflammatory cytokines. This can be achieved through targeted delivery of autoantigens, which can then expand or activate the regulatory immune cells, shifting the immune response from harmful pro-inflammatory to anti-inflammatory without triggering generalized immunosuppression [6,7,8,9].

In MS, peptides from myelin basic protein (MBP) or myelin oligodendrocyte glycoprotein (MOG) are used to induce tolerance. In type-1 diabetes (T-1D), insulin or glutamic acid decarboxylase 65 (GAD65) peptides are given in tolerogenic forms to promote immune tolerance. In RA, peptides derived from collagen or proteoglycan are formulated to induce regulatory immune responses. Unlike other therapeutic approaches, inverse vaccines aim to target specific autoimmune responses and provide potentially long-lasting disease remission by reestablishing immune tolerance, preserving overall immunity and inducing limited side effects.

As will be discussed below, a variety of tolerogenic pathways used by the immune system are being exploited to design inverse vaccine approaches that induce autoantigen-specific immune tolerance. They include suppression of the undesirable immune response through deviation, eradication, or inhibition of effector immune cells specific for the autoantigen, and even the initiation and/or growth of regulatory immune cells.

## 2. Regulatory T Cells and Maintenance of Immune Tolerance to Self

For both B cells and T cells, immune tolerance begins in early lymphocyte development in the bone marrow and the thymus, respectively, where autoreactive cells are either deleted or rendered tolerant to self-antigens. Regulatory T cells (Treg) can be categorized based on their cell surface markers, origin, and transcriptional expression. In the thymus, Treg development occurs via upregulation of cell surface proteins, i.e., the alpha unit of the IL-2 receptor (CD25), and requires cytokine signaling and expression of FOXP3, a transcription factor important for the functions of Tregs. In the periphery, generation of a regulatory phenotype requires stimulatory cytokines, such as IL-2 and TGF-β. Both Tregs that develop in the thymus (tTregs), and those that emerge in the periphery (pTregs) play a key role in suppressing inflammatory responses in peripheral organs [10,11].

Tregs exert their immunosuppressive effects via a number of mechanisms, including cytolysis, targeting of antigen presenting cells (APCs), and releasing inhibitory cytokines and small molecules. In addition to secreting factors that trigger activation of cell death pathways, Tregs produce cytokines that maintain an anti-inflammatory environment, such as IL-10 and TGF-β. Both T cells and APCs express CD80 and CD86 that act as co-stimulatory molecules. By contrast, CTLA-4 is constitutively expressed by Tregs and its expression on conventional T cells requires their activation. The immune checkpoint PD-1 also plays a role in promoting immune tolerance. PD-1-positive Tregs can interfere with autoreactive B cells via the PDL-1 receptor, thereby mitigating T cell activation [12].

In healthy subjects, Tregs play a key role in the control of autoreactivity and the maintenance of immune tolerance in the periphery [13]. However, in various autoimmune diseases, the frequency and/or suppressive functions of peripheral CD4^+^CD25^+^FOXP3^+^ Tregs are abnormal [14]. In principle, restoring self-tolerance could be achieved by selective induction of autoantigen-specific Tregs, as reported in several Treg-based immunotherapies [14,15]. For example, clinical trials showed that infusion of Tregs could represent a safe and effective treatment in patients with type-1 diabetes [16], and several ex vivo approaches for expanding Tregs have been described [14,15]. Currently, however, engineered immune cells are being used to tailor treatment strategies for human autoimmune diseases and other immune disorders [17].

## 3. Mechanisms That Shape the Functional Plasticity of Regulatory B Cells

Peripheral B cells can be identified by an ensemble of phenotypic cell surface markers, allowing their categorization into various subsets, including transitional/immature B cells, naive follicular B cells, germinal center B cells, memory B cells, and antibody-secreting plasma cells [18]. Conventional B cells play a key role in adaptive immunity, including antibody secretion, antigen presentation, and cytokine production. Additionally, marginal zone (MZ) B cells and B-1 cells exert innate-like functions [19,20,21].

The contribution of B lymphocytes to both innate and adaptive immune responses has been amply demonstrated through human studies and animal models [22]. In parallel to the well-established role of Tregs in autoimmune diseases, accumulating studies revealed the existence of B cell subsets with potent immunosuppressive and/or regulatory activities in homeostatic modulations [23]. Their functions have been investigated in autoimmune disorders, graft rejection, infection, and cancer. In humans, they were characterized as transitional B cells (CD19^+^CD24^hi^CD38^hi^), plasmablasts (CD19^+^CD27^int^CD38^+^), and Br1 cells (CD19^+^CD25^+^CD71^+^CD73^−^). Their numbers are reduced in patients suffering from autoimmune diseases.

Initially, the existence of a regulatory role of B cells (Breg) in autoimmunity was obtained using mouse models of MS, rheumatoid arthritis, and lupus [23]. Bregs have also been reported in clinical studies of B cell depletion therapy using anti-CD20 antibodies that target B cells from the pre-B cell to the memory stage, but not plasma cells [24]. For example, B cell depletion in MS patients resulted in beneficial outcomes, further supporting the pathogenic role B cells play in autoimmune disease [25]. However, anti-CD20 therapy also eradicates Bregs, potentially leading to disease exacerbation, as seen in colitis, or to the spontaneous ignition of colitis and psoriasis following B cell depletion [26,27,28,29]. Similarly, non-Hodgkin’s lymphoma patients treated with anti-CD20 antibodies also developed autoimmunity manifestations [30]. In mice suffering from experimental autoimmune encephalomyelitis (EAE), B cell deficiency achieved genetically (μMT) or by anti-CD20–depletion led to a reduction in the percentage of FOXP3^+^ Treg in the central nervous system [31,32,33]. In further studies, B cells were documented to regulate Treg expression by producing TGF-β and IL-10 [34].

The mechanisms underlying the regulatory role of B cells in maintaining immune tolerance to self-antigens have been addressed in several preclinical and human studies. In experimental studies, IL-10-producing Bregs can convert resting CD4^+^ T cells to Treg cells and suppress autoimmune diseases [35]. Strikingly, B cell-derived IL-35 promotes the conversion of B cells to a Breg subset that produces IL-10, and adoptive transfer of the resulting Bregs suppressed experimental autoimmune uveitis in mice with established disease by promoting Treg cell expansion [35].

In healthy individuals, human CD19^+^CD24^hi^CD38^hi^ B cells suppressed the differentiation of Th1 and Th17 cells as well as the release of TNF-α and INF-γ via production of IL-10 [36]. Moreover, these B cells converted CD4^+^CD25^−^ T cells into Tregs, also via IL-10 production. Importantly, the numbers of CD19^+^CD24^hi^CD38^hi^ B cells were reduced in the periphery of patients with autoimmune disease compared with healthy subjects [36], suggesting that, in autoimmune conditions, this regulatory B cell subset fails to prevent the emergence of autoreactivity and inflammation. In support of this view, experiments using an ex vivo co-culture model of autologous mixed human lymphocyte cultures showed that activated B cells regulate proliferation of T cells by producing TGF-β and indoleamine 2,3-dioxygenase, which triggers the induction of TGF-β-producing CD4^+^ T cells and IL-10-producing Tregs [37].

Overall, Breg therapy in mice has shown promising results in experimental models of MS, RA, T-1D, and lupus. These studies show that adoptive transfer of Bregs or in vivo induction of Bregs can reduce disease severity, shift immune balance toward regulation, and prevent or reverse tissue damage. Clinical efficacy studies in humans are promising. While no Breg-based therapies are approved, early-phase studies and indirect data provide insight. Targeted delivery or stimulation of IL-10-producing Bregs is under exploration, and small-scale studies or personalized cell therapies (tolerogenic B cell infusions) are in the early stages of development [23,38].

## 4. Tolerogenic Vaccination by Deprivation of Co-Stimulatory Signaling

Studies of immune tolerance showed that peripheral tolerance can be induced by deprivation of co-stimulatory signaling, allowing immune cells to become anergic or to convert to suppressive immune cells. For example, nanoparticles used as artificial synthetic APCs display epitopes on their surface, but are deprived of co-stimulatory molecules [39,40]. Since this approach is antigen-specific, it ensures that tolerization only takes place upon encounter with the specific antigen [41].

**Figure 1:**
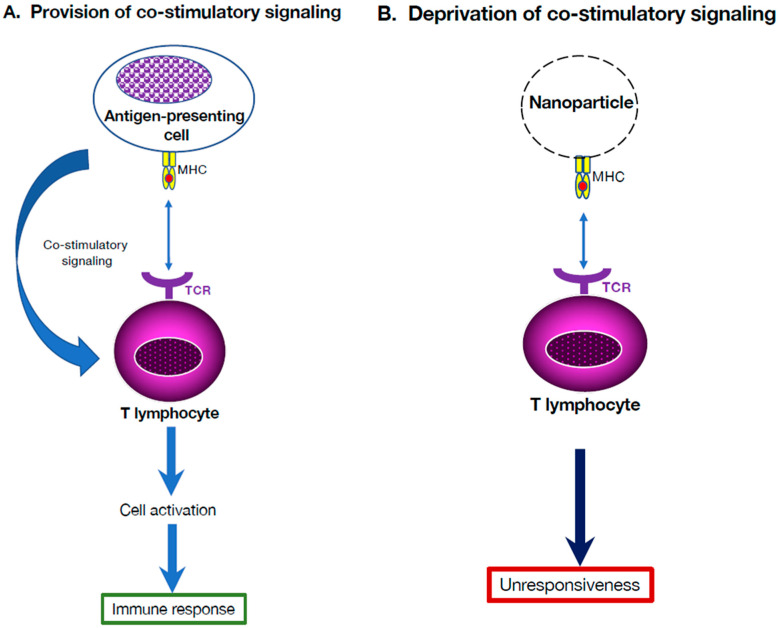
Tolerogenic vaccination by deprivation of co-stimulatory signaling. (**A**). In conventional immunization, the antigen is endocytosed and processed by antigen-presenting cells (APCs). It is then presented to T cells on MHC II molecules in the presence of co-stimulatory signaling. (**B**). In the absence of co-stimulatory signals, the antigen is directly displayed on MHC II molecules that decorate the nanoparticles used. In the absence of conventional APCs and co-stimulatory signaling, T cells recognize the antigen, but become anergic despite antigen recognition.

This approach has been tested in animal models of arthritis, type-1 diabetes (T-1D), and MS. For example, iron oxide nanoparticles displaying antigen-major histocompatibility complex (MHC) class II complexes allowed the induction of suppressive T cells and B cells [39,40]. Remarkably, infusion of these regulatory cells to naïve prediabetic animals achieved protection against diabetes development. Specifically, nanoparticles were decorated with MHC molecules, forming spike-like particles that are believed to mimic APCs’ ability to present epitopes to T cells (Figure 1). In a mouse model of T-1D, a nanoparticle carrying a single epitope reversed the disease symptoms, suggesting that the nanoparticles drive T cells to convert into regulatory T cells able to home to the inflammatory site and silence APCs, making them unable to activate T cells that perpetuate the disease [42]. Clinical trials are envisioned to test its applicability to autoimmune disease in humans.

## 5. Tolerogenic Vaccines Targeting the Liver Microenvironment

Initial observations showed that, through a combination of cytokines and tolerogenic APCs, the liver microenvironment favors immune tolerance to self [43]. Therefore, immunotherapeutic approaches that aim to restore or expand autoantigen-specific Treg activity might be beneficial for treating autoimmune diseases. In preclinical studies, ectopic expression of a neuronal autoantigen in the liver induced antigen-specific Tregs and suppressed autoimmune neuroinflammation [44], suggesting that targeting autoantigens to hepatocytes could represent a novel approach for autoimmune disease prevention or treatment. In studies of EAE, hepatic gene therapy with a vehicle harboring the DNA sequence encoding myelin oligodendrocyte glycoprotein (MOG) was found to induce the expansion of antigen-specific FOXP3^+^ Tregs, prevent disease development, and reverse preexisting EAE [45]. These observations suggest that Treg-mediated suppression of autoimmune disease can be achieved in vivo by taking advantage of the liver’s ability to promote immune tolerance.

**Figure 2:**
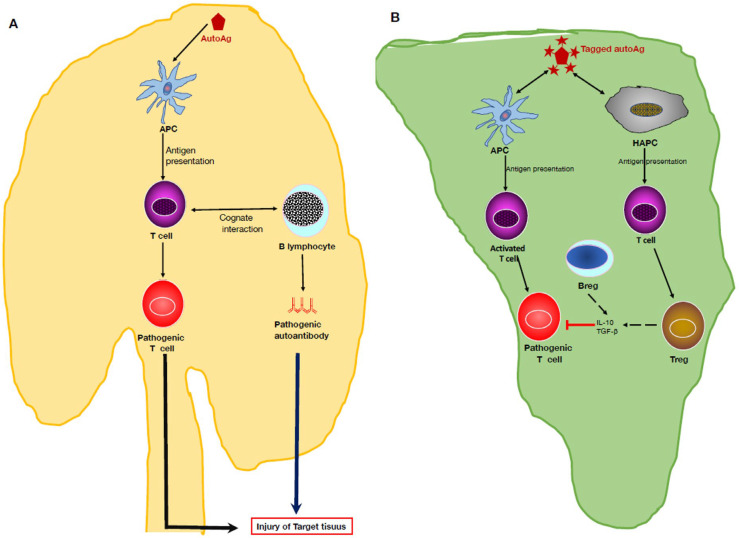
Induction of antigen-specific immune tolerance in the liver. (**A**). Traditionally, effective immunization with a native autoantigen is followed by antigen presentation to T cells by antigen presenting cells, i.e., dendritic cells and B cells, expressing co-stimulatory molecules. In secondary lymphoid organs, i.e., lymph nodes, cognate recognition results in T cell activation and differentiation into pathogenic effector T cells that release pro-inflammatory cytokines (i.e., IFN-γ and IL-17) which can, together with pathogenic autoantibodies, mediate tissue injury typical of autoimmune diseases. (**B**). In inverse vaccination, the autoantigen is tagged with residues, such as *N*-Acetylgalactosamine or *N*-Acetylglucosamine, that promote the selective accumulation of the autoantigen in the liver microenvironment. Then, hepatic antigen-presenting cells (HAPCs) present the autoantigen to T cells, which are thought to differentiate into Treg cells. As discussed in the text, both HAPCs and B lymphocytes express the co-inhibitory molecule programmed death ligand (PD-1), release TGF-β, activate Treg cells, which express PD-1 and GTIR, and release IL-10 and TGF-β. Bregs are also present in the liver, indicating that the dual generation of both Tregs and Bregs is what mitigates the activity of pathogenic autoreactive T cells and prevents tissue damage of the target tissues.

Further investigation disclosed that the homeostatic antigen-presenting potential of the liver microenvironment can result in tolerogenic education of T cells and induction of antigen-specific immune tolerance (Figure 2). In experimental studies, tagging antigens with N-acetyl-galactosamine (GalNAc) could trigger peripheral immune tolerance in the liver, leading to prevention of immune reactions to the nominal antigen. In the liver, hepatic antigen-presenting cells (HAPCs) express C-type lectins that exhibit promiscuity for GalNAc and *N*-acetylglucosamine (GluNAc). To amplify the antigen presentation potential of HAPCs and promote their capacity to induce lymphocyte tolerization, investigators produced multivalent polymeric synthetic GluNAc or GalNAc glycosylations to which antigens can be conjugated, allowing several HAPCs subsets to be targeted. In studies of a mouse model of T-1D, antigens modified with polymeric forms of GalNAc or GluNAc led to antigen-specific tolerance, expanded functional Tregs, and prevented diabetes mediated by activated diabetogenic T cells [46]. Mechanistically, antigen presentation to T cells in the presence of co-inhibitory signaling triggers a toleration program that changes their cellular fate and leads to exhaustion, anergy, death by apoptosis, or conversion to regulatory T cells expressing the FOXP3 transcription factor.

More recently, this therapeutic approach was shown to induce antigen-specific tolerance in EAE [9]. In inverse vaccination, the target autoantigen decorated with GalNAc is recognized as harmless in the liver, where immune cells tolerate and protect it instead of attacking it. In experimental models of MS, the inverse vaccine reversed the autoimmune injury and restored nerve functions. The conjugates used are preferentially taken up in the liver, thereby minimizing potential toxicities in other tissues. Transcriptional analysis revealed that the pathways involved were driven by PD-1 and the co-inhibitory ligand CD276 (B7-H3) [9]. However, the authors did not determine if their treatment platform prevented the induction of autoantibodies, which are known to contribute to disease progression in MS and other autoimmune disorders.

Celiac disease is caused by a combination of genetic factors and exposure to gluten. The only recommended therapy for this chronic autoimmune disorder remains a gluten-free diet. The safety and tolerability of a liver-targeting glycosylation signature (KAN-101) conjugated to a deaminated gliadin peptide were recently tested in a human phase 1 clinical trial [47]. The safety profile was acceptable in patients with celiac disease, but future trials should evaluate the efficacy of this therapeutic option in patients.

## 6. mRNA Vaccination, a Flexible Treatment Strategy for Autoimmunity

Vaccination has played a chief role in combating infectious diseases worldwide. Conventional vaccine platforms make use of attenuated or inactivated infectious agents, viral vectors, or antigen subunits, which often necessitate complex manufacturing processes. Recently, mRNA vaccines have been demonstrated to offer versatile platforms for rapid and specific vaccine development against emerging pathogens [48]. This technology harnesses the cellular machinery of the vaccinee to produce specific epitopes that can trigger an immune response specific for the immunogen, offering advantages over traditional vaccine approaches, as illustrated in the context of recent successes in the deployment of mRNA-based vaccines against the SARS-CoV-2 virus [49]. mRNA vaccines are frequently delivered by lipid-based nanoparticles exhibiting a potent transfection efficiency, a potential to trigger strong immune responses, and minimal undesirable effects. Beyond their application in preventing infectious diseases, the importance of nanoparticle mRNA vaccines holds promise for other medical challenges, including genetic disorders, personalized medicine, and cancer [50].

**Figure 3:**
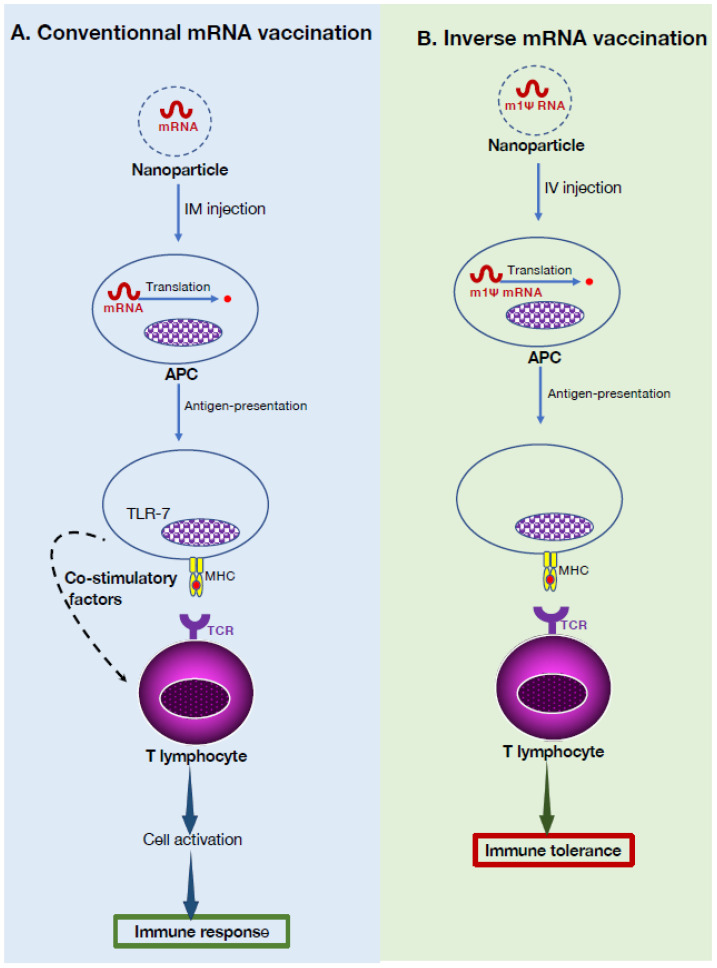
Mechanisms of mRNA-based tolerogenic vaccines. (**A**). In traditional vaccination against infectious agents, nanoparticles containing pro-inflammatory lipids and carrying a mRNA are injected intramuscularly to recruit local antigen-presenting cells (APCs). After uptake, the mRNA is recognized by ribosomes and translated into antigenic proteins. The resulting epitopes are presented to T cells in a pro-inflammatory environment, leading to their specific activation. This subsequently triggers activation of the immune response by secretion of inflammatory mediators. (**B**). In inverse vaccination, intravenous injection of non-inflammatory nanoparticles loaded with mRNA lacking danger signals (pathogen-associated molecular patterns) that can bind to pathogen recognition receptors (TLR7) results in induction of antigen-specific T cell tolerance. The nanoparticle carrying the mRNA coding for the tolerogenic antigen is first incorporated in APCs and, eventually, translated into the tolerogenic molecule. The resulting epitopes are loaded on MHC class II molecules and presented to naive T cells, which acquire a tolerogenic phenotype.

Recently, the technology of mRNA platforms was adapted to achieve antigen-specific immune tolerance for autoimmune disease treatment [51]. In conventional mRNA vaccination, the immunogen is encapsulated in nanoparticles exhibiting an inherent pro-inflammatory activity (Figure 3). Following intra-muscular injection, nanoparticles are taken up by APCs that have been activated by local pro-inflammatory signaling and TLR7 engagement through extracellular RNA derived from the nanovectors. Following homing to the lymph nodes, APCs will translate the mRNA to protein, and then, depending on the uptake route, present it on their MHC class I or MHC class II molecules. Eventually, APCs will encounter T cells specific to the immunogen in the lymph nodes, which will lead to successful T cell activation through appropriate APC-derived co-stimulatory signaling, together with pro-inflammatory factors. In the absence of co-stimulation, however, antigen presentation results in tolerization of antigen-specific T cells or even their deletion [52].

To achieve auto-antigen-specific immune tolerance, investigators used an experimental model of the autoimmune disease MS, namely EAE, triggered by immunization against myelin-derived auto-antigens [51]. Specifically, an mRNA encoding a myelin antigen was encapsulated in liposomes deprived of pro-inflammatory potential. A hallmark of this platform is that the nucleotide uridine in the mRNA was replaced with 1-methylpseudouridine (m1Ψ), rendering this m1Ψ mRNA capable of inducing protein translation but unable to recruit the TLR7 signaling pro-inflammatory pathway. This strategy had beneficial effects on EAE. It delayed or mitigated the severity of immune injury, but did not impair the overall potential of the immune system to respond to a new antigen [51]. Importantly, the m1Ψ mRNA used did not exacerbate levels of pre-existing autoantibodies in the rodents. Mechanistically, expression of the co-inhibitory receptors CTLA-4 and PD-1 on the effector and Treg cells was important for the effects observed [51]. If confirmed, this new-type of vaccination platform could represent a powerful and flexible novel strategy for autoimmune disease treatment.

In previous studies of mRNA COVID-19 vaccination, evaluation of the dynamic changes of antigen-specific humoral and cellular responses revealed that mRNA vaccination triggers an expansion of both Treg and Breg subsets [53]. It will be important to determine if the Breg subset is also recruited through mRNA vaccination for autoimmune diseases.

## 7. Liver-Resident B Lymphocytes

**Figure 4:**
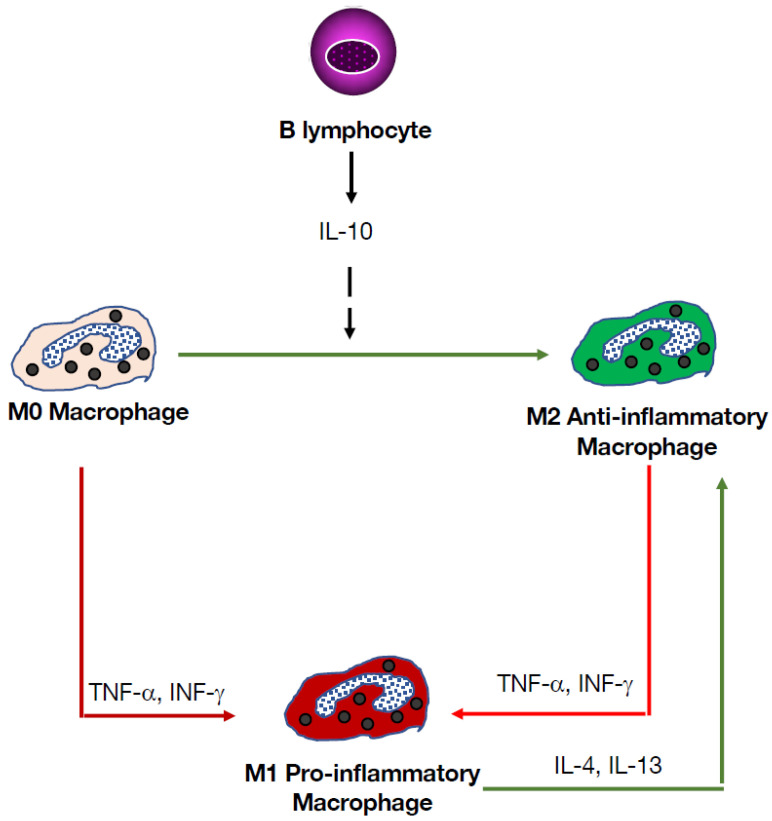
Liver-resident B cells that promote the emergence of anti-inflammatory macrophages. Non-activated M0 macrophages can potentially polarize into either pro-inflammatory M1 macrophages or mature anti-inflammatory M2 macrophages, which can further differentiate into M2a, M2b, M2c, or M2d macrophages upon stimulation with different cytokines. Subsequently, M2b macrophages can exert anti-inflammatory activities, and M2c macrophages are endowed with the ability to suppress immune responses. Recent studies indicate that IL-10 derived from B-2 cells can promote the emergence of anti-inflammatory macrophages [57].

The liver is known to exert several functions, including metabolism, protein synthesis, and clearance of undesirable metabolites and pathogens. These tasks require participation of liver-specific cells, such as hepatocytes, but also immune cells that include macrophages, T cells, NK cells, NKT cells, and B cells [54]. At the embryonic stage, the liver represents a site of origin for B cells that exhibit features of B-1 cells [55], distinct from those of the predominant population of B-2 cells. In the adult liver, B cells constitute a sizable proportion of the intrahepatic lymphocyte population [56]. They represent approximately 20% of lymphocytes in the liver [57]. Compared to B-2 cells, there are fewer B-1a cells in the liver (<25%) compared to the kidney, bladder, and lung (40%, 60% and 40%, respectively) [57]. Phenotypic and functional characteristics of intrahepatic B cells indicate that they are not of embryonic liver origin, but are probably derived from the bone marrow and, therefore, resemble conventional splenic B-2 cells. They are in an activated state, allowing them to act as effective APCs, promote T cell-mediated functions, and play a role in tissue repair [56]. Additionally, B cells produce cytokines and chemokines, enabling modulation of the extent and magnitude of the immune response [4]. They also are capable of both amplifying and suppressing immune responses. Following bacterial infection, murine B cells proliferate within the liver and undergo somatic hypermutation [58]. In humans, hepatitis infection also leads to B cell infiltration, including formation of tertiary lymphoid tissue and local germinal centers [59]. Importantly, intra-hepatic memory B cells express T-bet, CD80, and PD-L2, suggesting that the liver acts as a generative site of B cell responses during local infection, inflammation, and vaccination. Consistently, dysfunctional B cells have been implicated in the pathogenesis of liver diseases, including primary biliary cholangitis, autoimmune hepatitis, primary biliary cholangitis, and IgG4-related hepatobiliary disease [60]. More recently, the liver was found to house a population of extravascular, tissue-resident, IL-10-producing Bregs [57]. Importantly, intrahepatic Bregs, which are also present in humans, can have an impact on macrophage polarization (Figure 4) by promoting an anti-inflammatory phenotype via B cell-derived IL-10 [57].

## 8. A Tolerogenic Feedback Loop Implicating B Lymphocytes in Inverse Vaccination

Given the prominent role of CD4^+^FOXP3^+^ T regulatory cells in autoimmune disease, it is relevant to discuss the modulatory roles of B lymphocytes in inverse vaccination. In fact, the diverse and dynamic interactions between T cells and Bregs play a chief role in immune regulation. In early studies, Bregs could not only suppress the effector functions of T cells, but their interactions with T cells could also give rise to conventional Treg cells and IL-10-producing T cells [36,61]. In addition to IL-10, TGF-β represents a pleiotropic cytokine required for Treg-cell induction and function [62]. For example, Bregs were demonstrated to trigger immune tolerance through induction of Tregs via TGF-β production [63].

**Figure 5:**
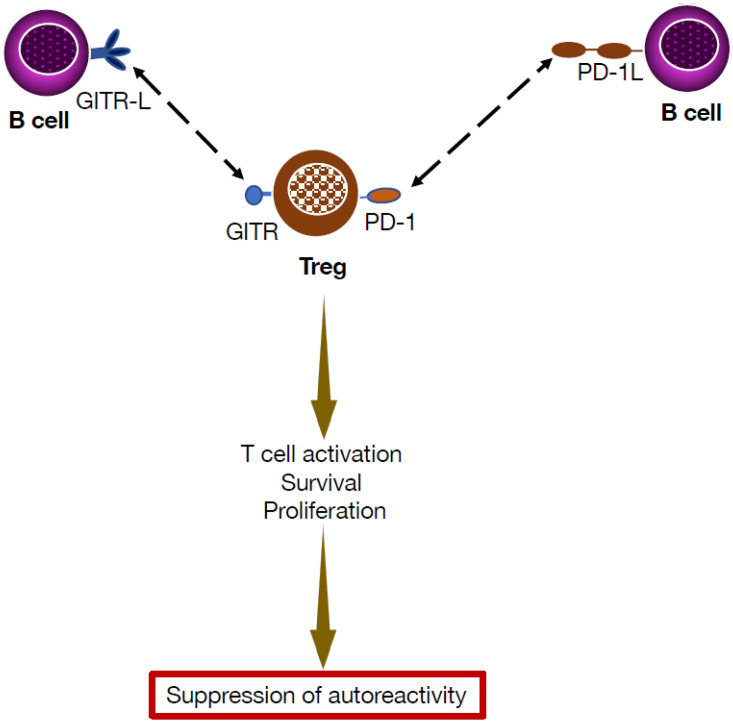
Model for T cell–B cell interactions that promote inverse vaccination in the liver microenvironment. In the liver microenvironment, activated Tregs express high levels of the checkpoint inhibitor PD-1 and glucocorticoid-induced tumor-necrosis-factor-receptor-related protein (GITR). Clustering of GITR can be induced by soluble or multivalent GITRL derived from B cells, which also express PD-1L. The downstream signaling following GITR multimerization and PD-1 engagement leads to Treg cell activation, and, consequently, immune suppression, which promotes inverse vaccination against autoreactivity.

Investigation of the homeostatic regulation of Treg fate in the periphery supports this contention. Glucocorticoid-induced TNFR family-related protein (GITR) is an immune receptor checkpoint that is constitutively expressed in Treg cells. Its engagement increases cell proliferation, and effector function. This receptor is expressed in dendritic cells and macrophages, but also in B cells [64]. The functional role for GITR in regulating CD4^+^CD25^+^ T cell expression has been demonstrated by showing that GITR is expressed on the surface of Treg cells [65,66], that its ligation by Fc-GITRL triggers Treg cell expansion [67], and that mice expressing a B cell-specific GITRL transgene manifest increased numbers of Tregs [64]. The observation that mice bearing a B cell-specific GITRL transgene exhibit increased numbers of peripheral FOXP3^+^ Tregs [64] suggested that B cells could be involved in nTreg homeostasis. In further studies of mice with EAE or colitis, B cell depletion was associated with a reduction in peripheral Treg cell numbers [68], indicating that B cells can modulate autoimmunity development through Treg regulations. Remarkably, B cell expression of GITRL stimulated Treg proliferation, thereby supporting the survival of peripheral Treg cells (Figure 5). Thus, these studies suggest that, via GITRL expression, B cells play a key role in maintaining Treg numbers above a threshold that enables prevention of autoimmunity [68]. In experiments showing that B cells can induce Treg proliferation in an IL-10-independent fashion [68], GITRL expression by B cells was essential, and the potential of B cells to modulate peripheral Treg expression and to resolve EAE could be abrogated by blocking GITRL on B cells [68].

In addition to GITRL, other co-stimulatory molecules play a role in Breg–Treg interactions, including PD-1 and its ligand PD-L1. Stimulation of B cells recruits the B cell receptor and negatively modulates B cell activation [69]. PD-1 is known to play a key role in Treg development and functions [70]. It is conceivable that PD-L1-expressing B cells can provide activating signals to PD-1-expressing Treg cells, leading to suppression of immune responses. An additional line of evidence of a PD-L1-mediated function of B cells comes from studies showing that PD-L1^High^ Bregs negatively modulate T-cell differentiation, and that transfer of PD-L1^High^ B cells could suppress EAE progression [71].

In the periphery, Tregs are crucial in the maintenance of immune tolerance, and their functions are orchestrated with those of Bregs and DCs. Tolerogenic DCs and Tregs can produce anti-inflammatory mediators (IL-10 and TGF-β) to foster an immune tolerant environment, and DCs must retain their immature state to attain tolerogenic potential. For their part, Bregs can modulate a tolerant state by directly promoting formation of Tregs via CD80/CD86 co-stimulation or by producing IL-10, TGF-β, and IL-35 [72,73,74]. Bregs can also produce IL-10 that acts on IL-10-receptors present on DCs, which leads to Treg expansion [75], suggesting a Breg–Treg–tolerogenic DC feedback loop in establishing a tolerant state. Additionally, Bregs have been documented to mediate an immunosuppressive role by direct inhibition of effector T cell functions [71].

## 9. Challenges Ahead

Current treatments for autoimmune diseases are based on drugs that suppress the immune system in order to mitigate inflammation and limit disease progression. However, side effects of the resulting suppressive state renders patients more prone to infections and other undesirable complications. Since inverse vaccines are designed to be highly targeted, they only suppress the particular immune response directed to a specific autoantigen present in the target tissue. Consequently, inverse vaccination is likely to result in fewer side effects and improved quality of life. Ideally, inverse vaccination could reprogram the immune system to tolerate the disruptive autoantigen and lead to long-term remission. Reminiscent of inverse vaccination, therapeutic vaccines aim to optimize immune homeostasis and modulate the immunopathological course of the disease. For example, CEL-4000, a peptide-based vaccine, represents a therapeutic approach that aims to reprogram the immune system, restoring tolerance and reducing information rather than merely suppressing symptoms. Its development could provide a new treatment option for patients with RA, particularly those who do not respond adequately to existing medications [76]. In addition to devastating autoimmune diseases, such as MS, T-1D, celiac disease, myasthenia gravis, and Graves’ disease, inverse vaccination could be applied to allergic diseases and even organ transplantation by educating the immune system not to attack the grafted tissue.

Inverse vaccines are in the early stages of development, and no drug is currently available on the market. Future research should address several issues. First, before the promising experimental studies of inverse vaccination can be translated into the bedside, we must identify the different cell types involved and gain insight into the mechanisms underlying the induction of immune tolerance. Second, the vaccine must be delivered to the relevant microenvironment, ensuring that it results in induction of immune tolerance, rather than immunity. Third, accurate identification of the specific autoantigen responsible for the autoimmune attack represents a major hurdle, particularly because some diseases, such as lupus and inflammatory bowel disease, involve multiple autoantigens and epitopes. Fourth, the extensive pre-clinical investigations and clinical trials required to determine the safety and effectiveness of inverse vaccines are likely to be long and expensive. Finally, like other novel drugs, potential long-term effects of inverse vaccines must be determined. Despite these significant challenges, the future of inverse vaccination appears promising, able to provide improvements for millions of patients.

## Data Availability

Not applicable.
